# The Expected and Unexpected Roles of Nitrate Transporters in Plant Abiotic Stress Resistance and Their Regulation

**DOI:** 10.3390/ijms19113535

**Published:** 2018-11-09

**Authors:** Guo-Bin Zhang, Shuan Meng, Ji-Ming Gong

**Affiliations:** 1 State Key Laboratory of Crop Biology and College of Agronomy, Shandong Agricultural University, Taian 271018, Shandong, China; gbzhang@sdau.edu.cn; 2 Southern Regional Collaborative Innovation Center for Grain and Oil Crops in China, College of Agronomy, Hunan Agricultural University, Changsha 410128, Hunan, China; mengshuan1987@126.com; 3 National Key Laboratory of Plant Molecular Genetics and CAS Center for Excellence in Molecular Plant Sciences, Shanghai Institute of Plant Physiology and Ecology, Chinese Academy of Sciences, Shanghai 200031, China

**Keywords:** nitrate transporter, abiotic stress, SINAR, NPF, nitrogen use efficiency

## Abstract

Nitrate transporters are primarily responsible for absorption of nitrate from soil and nitrate translocation among different parts of plants. They deliver nitrate to where it is needed. However, recent studies have revealed that nitrate transporters are extensively involved in coping with adverse environmental conditions besides limited nitrate/nitrogen availability. In this review, we describe the functions of the nitrate transporters related to abiotic stresses and their regulation. The expected and unexpected roles of nitrate transporters in plant abiotic stress resistance will also be discussed.

## 1. Introduction and Background

Nitrate is the major nitrogen for most terrestrial plants, which serves as both an essential nutrient and a signal molecule involved in plant metabolism, growth, development, and adaptation to various environments [1,2,3,4]. However, nitrate concentrations in the soil can vary by four orders of magnitude from the µM to mM range as nitrate ion is readily dissolved and very mobile in the soil [5]. Plants have evolved two nitrate uptake systems to counteract this fluctuation, including low-affinity transport system (LATS) and the high-affinity transport system (HATS). The absorbed nitrate is then transported to different parts of plants for further assimilation.

Diverse kinds of membrane proteins are found to be involved in absorption of nitrate from the external environment into plants, and its transportation and translocation between different parts of the whole plant. These transporters consist of NITRATE TRANSPORTER 1 (NRT1)/PEPTIDE TRANSPORTER (PTR) family (NPF), NRT2, CHLORIDE CHANNEL (CLC) family, and SLOWLY ACTIVATING ANION CHANNEL and their homologs (SLAHs). In addition to nitrate, they can transport a diverse range of substrates, allowing their participation in diverse biological processes including plant growth, development, and adaptation to complicated environments. Detailed and comprehensive information regarding these nitrate transporters have been reviewed [6,7]. However, more and more evidence shows that a large portion of them take part in plants’ response to adverse environmental conditions. In this review, we specifically focus on nitrate transporters involved in plant abiotic stress tolerance and their physiological roles and regulation in the related processes (Table 1).

## 2. The Function of Nitrate Transports Under Limited Nitrate/Nitrogen Conditions

Since nitrate transporters are mainly responsible for the uptake and transport of nitrate and limited nitrogen/nitrate condition occurs frequently, there are quite a few nitrate transporters coping with this abiotic stress.

### 2.1. NRT2 Family in Response to Nitrate/Nitrogen Limitation

The NRT2 proteins belong to high-affinity transport system which is responsible for transporting nitrate at low concentration and plays an important role in conditions with limited nitrogen [6,16]. To date, four of seven *Arabidopsis* NRT2 transporters have been shown to be involved in plant low nitrogen adaptation. Those four transporters are AtNRT2.1, AtNRT2.2, AtNRT2.4, and AtNRT2.5, with different spatio-temporal distributions in root [8,9,15,16,44]. *AtNRT2.1* is derepressed by nitrogen deprivation and is mainly localized in cortex cells of the root’s mature regions [8,9]. Under low nitrogen conditions, AtNRT2.1 mediates apoplastic nitrate absorption, IHATS (nitrate-inducible high-affinity transport system), and root system architecture [10,11,12,13]. It has been demonstrated that AtNRT2.1 was the major contributor to IHATS, because phenotypes related to IHATS were preferentially observed in *atnrt2.1* mutants and could be partially compensated by AtNRT2.2 [14]. Different from AtNRT2.1, AtNRT2.4 and AtNRT2.5 were responsible for nitrate uptake from soil into plants, and were induced in epidermal cells of young roots by long-term nitrogen starvation [8,15,16]. Using *nrt2.1 nrt2.2* double mutants and *nrt2.1 nrt2.2 nrt2.4* triple mutants, further comparative analyses showed that AtNRT2.4 contributed essentially to plant biomass production under low nitrate supply [15]. Moreover, researchers found that AtNRT2.5 was a key component along with AtNRT2.1, AtNRT2.2, and AtNRT2.4 for adult plants to cope with severe nitrogen starvation [8,16].

In rice, it has been shown that the partner protein OsNAR2.1 (nitrate assimilation related protein) was needed for the three plasma membrane localized nitrate transporters OsNRT2.1, OsNRT2.2, and OsNRT2.3a, and they worked together to absorb nitrate [18,19]. As the *osnar2.1* RNAi knockdown plants with synchronously suppressed expression of *OsNRT2.1*, *OsNRT2.2*, and *OsNRT2.3a* showed reduced plant growth and lower total nitrogen concentration under either low or high nitrate treatments compared with wide type plants [18], it was indicative that those three proteins were likely to be involved in ensuring optimal plant growth under various nitrate levels.

### 2.2. NPF Transporters in Response to Nitrate/Nitrogen Limitation

Besides the NRT2 proteins mentioned above, several NPF proteins also participate in low nutrient response. NRT2 proteins are exclusively responsible for nitrate uptake, while NPF proteins are involved in more transport processes such as nitrate distribution between different tissues.

The low-affinity nitrate transporter AtNPF7.3/AtNRT1.5 is localized on plasma membrane of root pericycle cells surrounding the xylem, and functions in xylem nitrate loading [45]. AtNPF7.3/AtNRT1.5 is required for suppressing the nitrate deficiency-induced leaf senescence, most likely by promoting foliar potassium accumulation [26,27]. Moreover, AtNPF7.3/AtNRT1.5 was proved to play an essential role in repressing leaf chlorosis and preventing growth retardation when external potassium was limited [28]. *AtNPF2.12*/*AtNRT1.6* is expressed in the vascular tissue of the silique and funiculus, and functions to deliver nitrate to the developing embryo. Disruption of AtNPF2.12/AtNRT1.6 reduces nitrate accumulation in seeds, and further leads to higher seed abortion rate. However, nitrate starvation treatment decreased the seed abortion rate of *npf2.12* mutants, indicating that the requirement of nitrate during early embryo development could be alleviated when external nitrogen was limited [31]. Furthermore, the study on AtNPF2.13/AtNRT1.7 revealed the details of nitrate remobilization from source leaves and elucidated its role in plant growth under nitrogen starvation conditions [32]. Expression of *AtNPF2.13*/*AtNRT1.7* was detected in the phloem of the leaf minor vein and was increased as leaves aged. However, AtNPF2.13/AtNRT1.7 hardly affects plant growth under nutrient sufficient conditions. Long-term nitrogen starvation retarded growth of *npf2.13* mutant plants, and one of the mutant lines even showed early senescence in older leaves when nitrogen was removed at reproductive stage [32]. Further study found that the AtNPF2.13/AtNRT1.7 modulating low nitrogen adaptation was negatively regulated by the protein ubiquitination pathway, which was mediated by NLA (Nitrogen Limitation Adaptation) [46].

OsNPF6.3/OsNRT1.1A, OsNPF6.5/OsNRT1.1B, OsNPF2.4, and OsNPF8.9b have been demonstrated to contribute to plant growth, yield production, and/or NUE under diverse nitrogen conditions, but with different underlying mechanisms. OsNPF6.3/OsNRT1.1A is a tonoplast-localized protein and was reported as a target to simultaneously manipulate rice yield and maturation. *OsNPF6.3*-overexpressing plants exhibited significantly improved growth, yield, and NUE under both low and high nitrogen conditions, mainly because of the upregulated expression of nitrogen utilization-related genes [38]. OsNPF6.5/OsNRT1.1B was reported to transport nitrate under both low and high nitrate concentrations and had an essential single-nucleotide polymorphism between *indica* and *japonica* rice. The *OsNPF6.5*/*OsNRT1.1B*-*indica* variation was shown to improve grain yield and NUE, probably by enhancing nitrate uptake and translocation and upregulation of nitrate response, especially when under relatively low nitrate conditions [39]. OsNPF2.4 is a low-affinity nitrate transporter involved in acquisition and long-distance transport of nitrate, including nitrate redistribution from old leaves to nitrogen-starved roots and young leaves. Dry weight of *npf2.4* mutants was always lower compared with the wide type plants under either normal or nitrate deficiency conditions [40]. *OsNPF8.9b* (refer to OsNRT1.1b to avoid confusion [7]) is one of the splicing forms of *OsNPF8.9*, encoding a protein with six transmembrane domains, but it showed nitrate transport activity when expressed in oocytes. Overexpression of *OsNPF8.9b* could increase nitrogen accumulation in plants and improve rice growth under various nitrogen conditions, including low nitrogen supply [41]. When overexpressed, *OsNPF8.20*/*OsPTR9* improved grain yield under the condition of no fertilizer or low ammonium supply [42], but the exactly underlying mechanism is still an open question.

## 3. Stress-Initiated Nitrate Allocation to Roots (SINAR) and Abiotic Stress

Once absorbed by plants, most nitrate, apart from the nitrate assimilated in roots or stored in vacuoles, is translocated to shoots, where carbon skeletons, energy, and reducing power derived from photosynthesis can be easily accessed and facilitate the conversion of inorganic nitrogen to organic nitrogen. Nitrate assimilation and photosynthesis are thus directly coupled, leading to efficient energy use in foliar chloroplasts, a process known as nitrate photoassimilation requiring long-distance root to shoot nitrate transport adopted by most herbaceous plants [47,48]. However, the ratio of nitrate between roots and shoots can be variable under different conditions, as determined by the process of long-distance nitrate transport and exerts a strong influence on NUE [49]. Previous studies and our research found that under adverse conditions plants tended to uptake less nitrate and transported less nitrate to shoots from roots, leading to more nitrate retained in roots, which we named as stress-initiated nitrate allocation to roots (SINAR) [29,30,33,50]. Until now, two nitrate transporters, AtNPF7.3/AtNRT1.5 and AtNPF7.2/AtNRT1.8, have been found to be mainly responsible for this process and the coordinated and precise regulation of their expression have also been extensively elucidated [29,30,33,45].

### 3.1. The Discovery of SINAR

Nitrate taken up into plants normally undergoes long-distance transport to aerial parts for further assimilation, though it is frequently observed that nitrate assimilation becomes prevalent in the roots when under various stress conditions such as low light intensity [48] and limited external nitrate availability [51,52]. This phenomenon is confusing since it reduces energy efficiency, which is not a behavior favorable to plant survival. Thus, the underlying physiological importance and regulating mechanisms keep attracting many scientists, but remain barely identified. About two decades ago, the distribution of nitrate in Cd^2+^-treated pea (*Pisum sativum*) plants were found to have been changed compared with untreated plants. More nitrate was retained in the roots and much less nitrate was allocated to the shoots of Cd^2+^-treated plants than control plants, resulting in a dramatic increase of nitrate ratio between roots and shoots. Inhibited transpiration leading to less nitrate transport to shoots and more nitrate accumulation in roots was initially proposed [50]. Nonetheless, the model could not properly explain why the ratio of potassium between roots and shoots was barely changed although the transport of both potassium and nitrate, through water flow driven by transpiration, was largely suppressed.

**Figure 1:**
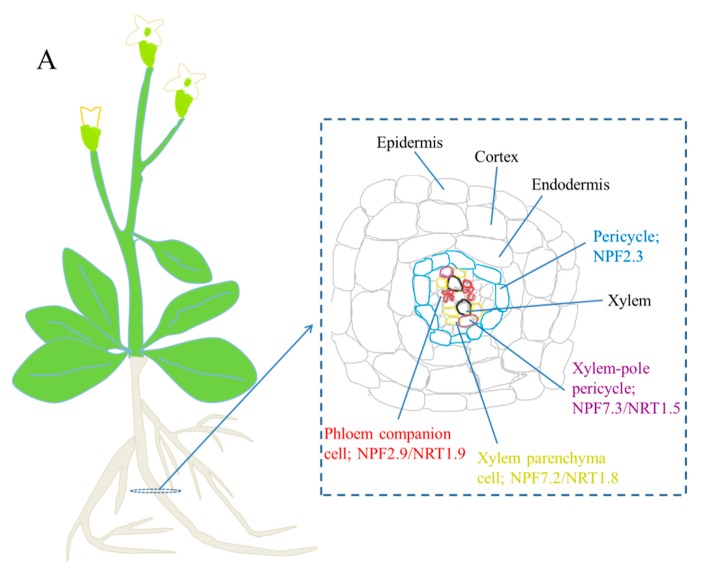

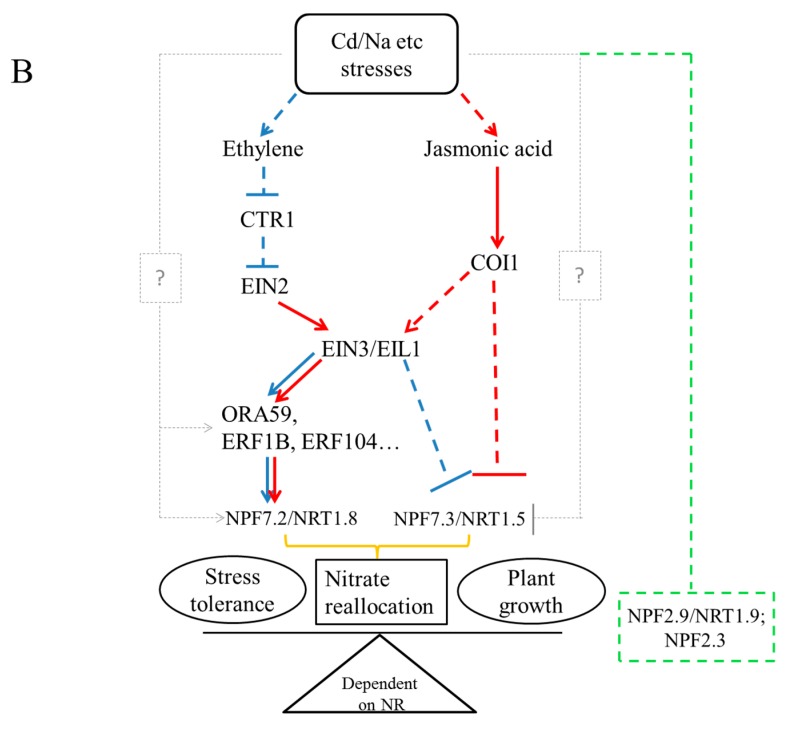
(**A**) The localization of nitrate transporters related to nitrate long-distance transport and (**B**) the regulation of stress-initiated nitrate allocation to roots (SINAR) by ethylene/jasmonate (ET/JA) signaling pathways to mediate plant adaptation to the environments. For (**B**), blue lines display the route for signals going through the ET signaling pathway, while red lines indicate those through the JA signaling pathway. Dashed lines indicate steps not shown or possible unidentified components. Gray dotted lines and question marks indicate alternative pathways/components that contribute, but to a much lesser extent, in the regulation of AtNPF7.3/AtNRT1.5 and AtNPF7.2/AtNRT1.8. In the stressed conditions, the expression of AtNPF2.3 and AtNPF2.9/AtNRT1.9 was unchanged, indicated by green lines and box. Note that (**A**) was revised from Figure 2 in [7] and (**B**) was revised from Figure 10 in [30].

Until 2010, the altered distribution of nitrate *in planta* was proved to be an active process rather than a passive outcome because a nitrate transporter AtNPF7.2/AtNRT1.8 was demonstrated to be actively involved in nitrate unloading from xylem vessels under cadmium stress. AtNPF7.2/AtNRT1.8 was initially identified based on its dramatic upregulation by Cd^2+^ stress in microarray experiments and hypothesized to transport oligopeptides chelating cadmium. However, based on the electrophysiological analyses using *Xenopus lacvis* oocytes, AtNPF7.2/AtNRT1.8 was demonstrated to be a low-affinity nitrate transporter. Several pieces of evidence were believed to support that the reallocation of nitrate to roots was actively regulated by AtNPF7.2/AtNRT1.8 (Figure 1A) (1) The transcript level of *AtNPF7.2*/*AtNRT1.8* was upregulated within a very short time; (2) The nitrate concentration in the xylem sap of AtNPF7.2/AtNRT1.8-null plants treated with cadmium was higher than treated wild-type plants; (3) The *npf7.2* mutants were more sensitive to cadmium stress under high nitrate conditions than the wild-type plants. These observations suggested that under cadmium stress, the expression of *AtNPF7.2*/*AtNRT1.8* was induced and a large amount of nitrate was removed from root xylem vessels leading to the decrease of nitrate concentration in the xylem sap. Coupled with the reduction of nitrate transport rate due to suppressed transpiration it further inhibited sap flow resulted in the dramatic decrease of nitrate content of shoots [33].

In addition, AtNPF7.3/AtNRT1.5, a xylem nitrate-loading transporter (Figure 1A) [45], was proved to be deeply involved in the regulation of nitrate reallocation conferring plants stress tolerance. Salt, drought, and cadmium stress could repress the expression of *AtNPF7.3*/*AtNRT1.5* and the null mutants of *AtNPF7.3*/*AtNRT1.5* showed enhanced tolerance to these stresses [29]. In addition to those, microarray data showed that many other abiotic stresses (such as osmotic, cold) and biotic stresses (such as *Pseudomons syringae*) could simultaneously induce the *AtNPF7.2*/*AtNRT1.8* expression and suppress *AtNPF7.3*/*AtNRT1.5* expression [29,33]. Given that the opposite expression of both genes was highly coordinated and occurred under diverse adverse conditions, the SINAR was believed to be modularized and universal.

### 3.2. Ethylene/Jasmonic Acid-NRTs Signaling Modulates SINAR

The findings that many different stresses (biotic or abiotic) could activate the coordinated expression of *AtNPF7.3*/*AtNRT1.5* and *AtNPF7.2*/*AtNRT1.8* led to the hypothesis that there must exist some common mechanism fine-tuning their opposite expression. In the search of immediate upstream factors of *AtNPF7.2*/*AtNRT1.8*, the roles of ethylene (ET) signaling pathway and jasmonate (JA) signaling pathway played in the regulation of the expression of *AtNPF7.3*/*AtNRT1.5* and *AtNPF7.2*/*AtNRT1.8* were well elucidated, by multidisciplinary approaches of genetics, molecular biology, and biochemistry [30]. In brief, stresses such as cadmium and salt stimulate the generation of ET and JA, which further activates the ET and JA signaling pathways, respectively. The signals transduced by ET and JA cascades are convergent on EIN3/EIL1 transcription factors, which further activate the expression of *ERFs*. ERFs induce *AtNPF7.2*/*AtNRT1.8* expression by binding to its promoter; meanwhile, EIN3/EIL1 suppresses *AtNPF7.3*/*AtNRT1.5* expression through decoding signals via the ET pathway and binding to its promoter. Note that the JA pathway also participates in the downregulation of *AtNPF7.3*/*AtNRT1.5* via COI1, yet independent of EIN3/EIL1. The coordinated upregulation of *AtNPF7.2*/*AtNRT1.8* and downregulation of *AtNPF7.3*/*AtNRT1.5* enhance nitrate removal from xylem vessels and lessen nitrate loading into xylem vessels simultaneously, resulting in SINAR. Thus, the ET/JA-NRT signaling module serves as a primary mechanism to mediate the crosstalk between SINAR and environment [30,45,53,54].

In Zhang’s study [30], a mutant *ein2-50 coi1-1*, in which both ET and JA signaling are essentially blocked, was generated for further research. In *ein2-50 coi1-1*, the expression of *AtNPF7.3*/*AtNRT1.5* and *AtNPF7.2*/*AtNRT1.8* could only be slightly influenced by stresses, which indicated that the transcriptional regulation of both genes was mainly through ET/JA signaling, whereas other signaling pathways pertaining to SINAR likely played a minor role. Meanwhile, the SINAR was almost blocked in the mutant, indicating that SINAR was predominantly modulated by ET/JA signaling pathways and the contribution of the other related signaling pathways to SINAR was negligible (Figure 1B). Other nitrate transporters mediating long-distance nitrate transport may also be involved in SINAR. However, it is certain that these transporters were either regulated by ET/JA signaling, or independent of ET/JA signaling pathway but with subtle contribution to SINAR [30]. Indeed, the nitrate transporter AtNPF2.3, a member of the NAXT (nitrate excretion transporter) sub-group of the NPF and constitutively expressed in root pericycle cells (Figure 1A), was demonstrated to contribute to nitrate translocation to the shoots under salinity, but when or how it contributed to SINAR remains to be determined [34].

The allocation of nitrate between roots and shoots is not only through xylem but also phloem. AtNPF2.9/AtNRT1.9, a low-affinity plasma membrane nitrate transporter expressed in the companion cells of root phloem (Figure 1A), participates in loading of nitrate into the root phloem and may facilitate shoot-to-root nitrate transport [55]. However, under any stresses or stress hormones, the *AtNPF2.9*/*AtNRT1.9* expression was rarely altered [56]. This observation together with the AtNPF2.3 study suggested that there was a great chance that *AtNPF7.3*/*AtNRT1.5* and *AtNPF7.2*/*AtNRT1.8*, among all the identified and characterized nitrate transporters responsible for long-distance nitrate transport (Figure 1B), were the only two nitrate transporters transcriptionally responsive to stresses.

### 3.3. SINAR Balances Plant Growth and Stress Tolerance

SINAR could impact on the stress tolerance of plants. The mutant plants with impaired SINAR are more sensitive to stresses such as cadmium and salt, whereas the mutant plants with enhanced SINAR show enhanced tolerance to stress. A lot of studies demonstrated that ET or JA signaling pathway regulated plant stress tolerance and quite a few important factors involved were also identified [53,57,58,59,60]. When changing nitrate concentrations in growth media, SINAR was introduced to ethylene-insensitive or jasmonate-insensitive mutants, which allows comparing the growth rate of various mutants under stressed conditions. The authors verified that SINAR is not only genetically downstream of ET/JA signaling but also contributing to the stress tolerance mediated by ET/JA signaling.

Under unstressed conditions, the mutant plants with mimicked SINAR (constitutively increased nitrate accumulation in the *npf7.3* roots without stress treatment) showed inhibited root growth in high nitrate media. Besides, *ctr1-8* with constitutive ethylene response and *eto1-1* with over-produced ethylene also showed similar phenotypes corresponding with external nitrate concentration. Furthermore, the inhibited root growth was partially restored when SINAR was lessened by crossing them with *nrt1.8-2*. All those results implied that the inhibition of root growth typical of plant response to ET or JA was also partially attributable to SINAR [30]. Note that AtNPF7.3/AtNRT1.5 also participates in coordinating nitrate and potassium signaling pathways, and further balance plant growth and leaf senescence. However, the contribution of SINAR to this process is still not clear [26].

Combining the findings above, a clear pattern about SINAR is found as follows: Mimicked SINAR would suppress plant growth under unstressed conditions, while under adverse conditions the plants with enhanced SINAR are more tolerant of stresses, which means that SINAR could regulate the trade-off between plant growth and stress tolerance. Since diverse abiotic and biotic stresses could trigger ET or JA signaling and subsequently activate SINAR, SINAR might serve as a universal and dynamic adaptive response of plants under diverse conditions and confer plants more flexible ability of dealing with varied ambient environments (Figure 1B).

### 3.4. The Downstream Events of SINAR

In the initial studies, the altered allocation of nitrate between roots and shoots was found to lead to more cadmium or sodium retained in roots, which did less harm to the shoots and improved plant adaptation to these stresses [50]. However, exactly how SINAR regulates the allocation of sodium or cadmium and further promotes stress tolerance remains largely illusive [29,33]. Nitrate retained in the roots was considered as a signal activating its downstream pathway in the first place [1,3,7,61]. However, unexpectedly, when nitrate reductase (NR) activity was almost blocked, all the plants responded to Na/Cd treatments similarly and showed similar growth rate no matter to what extent the SINAR was altered. When nitrate could not be reduced, it was supposed to function only as a signal molecule, which together with the above results suggested that SINAR functioned dependent on NR activity, and nitrate per se served as a signaling molecule involved in the SINAR-mediated plant environmental adaptation [30].

Nitrate could be reduced to nitrite, and further be reduced to nitric oxide (NO) by NR. NO has been established as a signaling molecule of significant importance, regulating plant growth, development, and stress adaptation [62]. However, application of the NO scavenger 2-(4-carboxyphenyl)-4,4,5,5-tetramethylimidazoline-1-oxyl-3-oxide (cPTIO) in the media did not alter the SINAR-related response under various conditions, suggesting that NO might not be associated with SINAR [30]. However, we currently do not know if other nitrogen assimilates including nitrite, ammonium, amino acids, and further downstream products, get involved in the SINAR signaling process and are possibly responsible for those phenotypes observed, mainly because we do not have evidence yet to exclude that possibility. Moreover, these chemicals are reduced from nitrate dependent on the NR reductase, and many of them affect cellular metabolism directly or serve as potential signal molecules in diverse signaling pathways [2,62,63]. One supportive evidence is the altered accumulation of proline and malondialdehyde in *npf7.3* plants [29], and proline has been established to have multiple roles in protein synthesis, osmotic protection, redox homeostasis and even signaling molecule mediating mitochondrial stress relief and development.

NR activity *in planta* is precisely and strictly regulated at diverse levels. AtSIZ1, an E3 SUMO ligase, regulates nitrogen assimilation in *Arabidopsis* through sumoylating NIA1 and NIA2, two nitrate reductases, and enhancing their activity. NR activity was reduced in the mutant *siz1-2* and the mutants displayed a dwarf phenotype, early flowering and abnormal seed development. Surprisingly, the salicylic acid (SA) levels was higher in *siz1-2* and the expression of the pathogenesis-related genes *PR1* and *PR2* were thus induced consistently with enhanced disease resistance to bacterial pathogens, and exogenous ammonium application could restore almost all those phenotypes [64]. These observations indicated that the affected nitrate assimilation could exert influence on the biosynthesis of SA and disease resistance, yet the underlying mechanism of this is largely unknown, giving us a hint that NR-dependent SINAR may function in similar ways and play an important role in the trade-off between growth and environmental adaptation through nitrate metabolism.

### 3.5. SINAR Engineering as A New Target of NUE Improvement

Since nitrate assimilation is supposed to be highly efficient only when it is coupled with foliar photosynthesis, and the nitrate distribution between root and shoots affect plant adaptation to environments, SINAR, serving as a sensitive response and machinery, thus evolved in plants. Now, with the development of gene-editing techniques and more comprehensive understanding of SINAR, genetic engineering of SINAR is becoming feasible [65,66]. In general, the nitrate taken up needs to be transported to shoots as much as possible under favorable environments, whereas SINAR should be induced sooner and more robustly to help plants deal with stresses. A most appropriate nitrate ratio between roots and shoots fits the criterion of best trade-off between plant growth and stress tolerance, though quantification of such a ratio still requires extensive experiments under diverse conditions.

The engineering of Na^+^ exclusion from the shoot has been achieved using an enhancer trap expression system for strong overexpression of *HKT1;1* specifically in the mature root stele [67]. We could achieve the targeted overexpression of *AtNPF7.3*/*AtNRT1.5* or *AtNPF7.2*/*AtNRT1.8* in a similar way. Alternatively, more GCC boxes could be introduced into *AtNPF7.2*/*AtNRT1.8* promoter or more EBSs (EIN3-binding sites) could be introduced into *AtNPF7.3*/*AtNRT1.5* promoter on the basis of our current knowledge in order to confer plants with a stronger response to stresses [30,53,54,60]. Besides, some specific amino acid sites responsible for nitrate transport could be precisely edited to enhance the transport capacity of AtNPF7.3/AtNRT1.5 or AtNPF7.2/AtNRT1.8 or modify the cellular polarized expression of these transporters, leading to more nitrate flow involved in SINAR [68,69,70,71]. Certainly, the ET/JA signaling module could also be elaborately designed to engineer SINAR [30,53,54,58,59,60,72,73,74].

Vacuolar sequestration capacity (VSC) of nitrate is associated with NUE [7,49]. Recently, two *Brassica napus* cultivars with high or low NUE were characterized and the different NUE was proved to be associated with nitrate VSC and long-distance nitrate transport [48,75,76,77]. The authors proposed that a decrease in root VSC of nitrate would enhance nitrate transport to shoots and promote NO_3_^−^ allocation to aerial parts, likely through coordinated regulation of *BnNRT1.5* and *BnNRT1.8*, thus contributing to higher NUE. This study not only gives us more detailed information about how long-distance nitrate transport is regulated, but also provides another line of evidence supportive of editing SINAR to improve NUE.

## 4. Other Nitrate Transporters in Response to Abiotic Stress

Despite the above-mentioned nitrate transporters and abiotic stress, there are a few other nitrate transporters involved in different kinds of abiotic stress. Nevertheless, the underlying mechanisms of them conferring plants resistance to abiotic stress are still largely unknown.

### 4.1. AtNPF6.3/AtNRT1.1/CHL1 and Abiotic Stress Tolerance

AtNPF6.3/AtNRT1.1/CHL1 plays the most important role in nitrate uptake and signaling, which displays dual affinities for nitrate and is also a transceptor perceiving the external nitrate concentration [68]. Besides, with several mutants exploited and phenotype characterization, AtNPF6.3/AtNRT1.1/CHL1 has been demonstrated to be related to many different kinds of abiotic stress.

Nitrate uptake in roots is largely attributable to AtNPF6.3/AtNRT1.1/CHL1, a symporter that could simultaneously transport one nitrate ion and two protons to root cells from soil or medium. Thus, it was proposed that AtNPF6.3/AtNRT1.1/CHL1 might be involved in plant tolerance to proton toxicity. Using the *npf6.3* knockout mutants and another nitrate uptake- and sensing-decoupled mutant *chl1-9*, functional investigation showed that these mutants had reduced proton tolerance in comparison with the wild-type plants, and nitrate uptake activity was required for the AtNPF6.3-conferred proton tolerance. This function was due to the direct effect of AtNPF6.3/AtNRT1.1/CHL1 nitrate transport activity [21].

Besides, the indirect effect of AtNPF6.3/AtNRT1.1/CHL1 nitrate transport activity was also found. The loss of AtNPF6.3/AtNRT1.1/CHL1 functional mutants under cadmium treatment was found to gain more biomass, accumulate less cadmium and several other metals in both roots and shoots in the presence of nitrate, whereas no difference was observed between the mutant and the wild-type plants in the absence of nitrate. These results suggested that functional disruption of AtNPF6.3/AtNRT1.1/CHL1 inhibited cadmium uptake, thus enhancing cadmium tolerance dependent on NO_3_^−^ uptake activity [23]. In addition, Na^+^ accumulation was also partially defective in *npf6.3* mutants, suggesting that AtNPF6.3/AtNRT1.1/CHL1 either partially mediated or modulated the nitrate-dependent Na^+^ transport [20].

Although *AtNPF6.3*/*AtNRT1.1*/*CHL1* is exclusively expressed in the tips of primary and lateral roots, young leaves, and developing flower buds, strong *AtNPF6.3*/*AtNRT1.1*/*CHL1* expression was also found in guard cells of mature leaves and hypocotyls. Furthermore, when grown in light or deprived of CO_2_ in the dark, *npf6.3* mutants had reduced stomatal opening and transpiration rates, leading to enhanced drought tolerance of *npf6.3* mutants compared with the wild-type plants. It was proposed that during stomatal opening, less nitrate was accumulated in the guard cells of *npf6.3* mutants and nitrate-induced depolarization of guard cells was impaired. These observations suggest that AtNPF6.3/AtNRT1.1/CHL1’s function in drought tolerance needs the presence of nitrate [22].

### 4.2. Nitrate Transporters and Hormone Abscisic Acid (ABA)

ABA is a phytohormone involved in the regulation of plant development and plant adaptation to adverse stress conditions [78]. Several ABC (ATP-binding cassette) transporters were identified to transport ABA using mutant analysis and biochemical characterization [79]. More recently, several NPF family members were also identified as ABA-importing transporters (AIT1-AIT4), using an improved yeast two-hybrid screen. Further transport assays demonstrated that AIT1, also known as AtNPF4.6/AtNRT1.2, mediated cellular ABA uptake. A lower surface temperature of inflorescence stems was observed in *ait1* mature plants compared with the wild-type plants, indicative of wider stomatal aperture in *ait1* mutants. These data suggested that AtNPF4.6/AtNRT1.2 functions as an ABA importer to regulate the stomatal aperture in shoots. So it is highly possible that this transporter gets involved in the response to drought stress [24]. Since the NPF family transports nitrate, further research should determine whether interactions exist between nitrate signaling/nutrition and ABA or even stress tolerance.

Recently, a large number of NPFs capable of translocating ABA, GA, and/or JA-Ile were identified [80], among which NPF3.1 could transport ABA and GA in vitro. In vivo experiments showed that ABA promoted *NPF3.1* expression and GA level in plants, indicating that GA–ABA interaction might occur at the level of transport and were involved in the downstream physiological processes [37]. However, the comprehensive functions of most NPFs identified to transport ABA, GA, and/or JA-Ile remain to be elucidated.

### 4.3. Other Transporters Involved in Abiotic Stress Resistance

In addition to the transporters mentioned above, several other transporters may also be involved in abiotic resistance. For example, AtNPF2.4 catalyzed passive Cl^−^ efflux out of cells and was much less permeable to NO_3_^−^. Shoot Cl^–^ accumulation decreased in the *atnpf2.4* knockdown mutants and overexpression of AtNPF2.4 led to increased shoot Cl^–^ accumulation, indicating that AtNPF2.4 might function to load Cl^–^ into the xylem of *Arabidopsis* roots during salinity stress [35]. Another Cl^–^ permeable transporter AtNPF2.5, the closest homolog to AtNPF2.4, was also demonstrated to function as a pathway for Cl^–^ efflux from the root, contributing to exclusion of Cl^–^ from the shoot of *Arabidopsis* under salt stress [36].

Besides, two mutants with point mutations in AtNPF6.4/AtNRT1.3 were isolated and observed to show increased polyamine resistance, suggesting that nitrate transport is closely related with polyamine transport or metabolism in parenchymal tissue of *Arabidopsis* shoots [25]. In addition, ROS accumulation was found to be accompanied with elevated *AtNRT2.6* expression in response to the redox-active herbicide methyl viologen, indicative of a possible relationship between AtNRT2.6 activity and the production of ROS regarding abiotic stress [17].

Moreover, intensive investigation of anion/proton exchanger AtCLCa, a nitrate transporter responsible for accumulating anions in the vacuole during stomatal opening, also mediated anion release during stomatal closure upon the application of stress hormone ABA [43]. Since stomatal opening/closing was closely related with drought stress tolerance, AtCLCa was most likely involved in the response to drought stress.

## 5. Conclusions

Although nitrate transporters were believed to be preferentially responsible for nitrate uptake and translocation and supposed to be closely related with adaptation to nitrogen/nitrate availability, many other unexpected roles related to abiotic stress have been found, including SINAR, phytohormone transport, and other unknown roles yet to be determined. These findings led us ask why even one nitrate transporter plays more than one physiological role. Nitrates impact plant metabolism, growth, and development, but how they link plant growth and adaptation to environments remains an open question. Luckily, cases such as SINAR have been well elucidated, which allows us to modify some specific transporters to achieve promising breeding goals.

## Figures and Tables

**Table 1:** Summary of nitrate transporters involved in abiotic stress response.

Gene	Locus Tag	Species	Function in Abiotic Stress Response	Reference
**Nitrate Transporter 2 *(NRT2)* Family**				
*AtNRT2.1*	*At1g08090*	*Arabidopsis thaliana*	Absorption of nitrate; contributes to IHATS; plays roles in root system architecture under low nitrogen condition	[8,9,10,11,12,13,14]
*AtNRT2.2*	*At1g08100*	*Arabidopsis thaliana*	Partially compensates *AtNRT2.1* under low nitrogen treatment when *AtNRT2.1* was disrupted	[12,13,14]
*AtNRT2.4*	*At5g60770*	*Arabidopsis thaliana*	Contributes to plant biomass production under low nitrate supply	[8,15]
*AtNRT2.5*	*A1g12940*	*Arabidopsis thaliana*	Promotes adult plants to cope with severe nitrogen starvation	[8,16]
*AtNRT2.6*	*At3g45060*	*Arabidopsis thaliana*	Probable link between NRT2.6 activity and the production of ROS (reactive oxygen species) in response to abiotic stress	[17]
*OsNRT2.1* *OsNRT2.2* *OsNRT2.3a*	*Os02g02170* *Os02g02190* *Os01g50820*	*Oryza sativa*	Likely to work together on reducing suppression of plant growth when nitrate is deficient	[18,19]
**Nitrate Transporter 1 *(NRT1)*/Peptide Transporter *(PTR)* Family *(NPF)***				
*AtNPF6.3*/*AtNRT1.1*/*CHL1*	*At1g12110*	*Arabidopsis thaliana*	Involved in plant tolerance to proton toxicity; inhibition of AtNPF6.3 activity reduces cadmium uptake; plant Na^+^ accumulation was also partially defective in *npf6.3* mutants; function in drought tolerance in the presence of nitrate	[20,21,22,23]
*AtNPF4.6*/*AtNRT1.2*/*AIT1*/	*At1g69850*	*Arabidopsis thaliana*	Imports hormone abscisic acid (ABA) and important for the regulation of stomatal aperture in shoots	[24]
*AtNPF6.4*/*AtNRT1.3*/	*At3g21670*	*Arabidopsis thaliana*	Polyamine resistance is increased in *npf6.4* mutants	[25]
*AtNPF7.3*/*AtNRT1.5*	*At1g32450*	*Arabidopsis thaliana*	Downregulated by cadmium, salt stress, and knockout mutants show enhanced tolerance to abiotic stress; Suppress leaf senescence under nitrate deficiency and repress leaf chlorosis and growth retard when external potassium was limited	[26,27,28,29,30]
*AtNPF2.12*/*AtNRT1.6*	*At1g27080*	*Arabidopsis thaliana*	Positively regulates seed abortion rate under nitrogen starvation	[31]
*AtNPF2.13*/*AtNRT1.7*	*At1g69870*	*Arabidopsis thaliana*	Inhibits plant growth retardation upon nitrogen starvation	[32]
*AtNPF7.2*/*AtNRT1.8*	*At4g21680*	*Arabidopsis thaliana*	Upregulated by cadmium, salt stress and knockout mutants show enhanced sensitivity to abiotic stress;	[30,33]
*AtNPF2.3*	*At3g45680*	*Arabidopsis thaliana*	Contributes to nitrate translocation to the shoots under salinity	[34]
*AtNPF2.4*	*At3g45700*	*Arabidopsis thaliana*	Regulation of Cl^–^ loading into the xylem of *Arabidopsis* roots during salinity stress	[35]
*AtNPF2.5*	*At3g45710*	*Arabidopsis thaliana*	Modulates chloride (Cl^−^) efflux from roots of *Arabidopsis*	[36]
*AtNPF3.1*	*At1g68570*	*Arabidopsis thaliana*	Transport ABA and GA (gibberellic acid) in vitro	[37]
*OsNPF6.3*/*OsNRT1.1A*	*Os08g05910*	*Oryza sativa*	Improves rice growth, yield and NUE (nitrogen use efficiency) under both low and high nitrogen conditions when overexpressed	[38]
*OsNPF6.5*/*OsNRT1.1B*	*Os10g40600*	*Oryza sativa*	*OsNPF6.5*/*OsNRT1.1B*-*indica* variation was shown to improve grain yield and NUE under diverse nitrogen conditions	[39]
*OsNPF2.4*	*Os03g48180*	*Oryza sativa*	Contributes to plant growth under either normal or nitrate deficiency conditions	[40]
*OsNPF8.9*	*Os03g13274*	*Oryza sativa*	Over-expression of *OsNPF8.9b* improves plant growth under low nitrogen (contain nitrate and ammonium) treatment	[41]
*OsNPF8.20*/*OsPTR9*	*Os06g49250*	*Oryza sativa*	When overexpressed, *OsNPF8.20* improved grain yield under the condition of no fertilizer or low ammonium supply	[42]
**Chloride Channel (*CLC*) family**				
*AtCLCa*	*At5g40890*	*Arabidopsis thaliana*	Accumulating anions in the vacuole during stomatal opening but also mediated anion release during stomatal closure in response to the stress hormone abscisic acid (ABA)	[43]
